# Compensatory changes in cortical resource allocation in adults with hearing loss

**DOI:** 10.3389/fnsys.2013.00071

**Published:** 2013-10-25

**Authors:** Julia Campbell, Anu Sharma

**Affiliations:** ^1^Department of Speech, Language and Hearing Sciences, University of Colorado at BoulderBoulder, CO, USA; ^2^Institute of Cognitive Science, University of Colorado at BoulderBoulder, CO, USA

**Keywords:** adult, sensorineural hearing loss, cortical auditory evoked potential, cortical resource allocation, source localization

## Abstract

Hearing loss has been linked to many types of cognitive decline in adults, including an association between hearing loss severity and dementia. However, it remains unclear whether cortical re-organization associated with hearing loss occurs in early stages of hearing decline and in early stages of auditory processing. In this study, we examined compensatory plasticity in adults with mild-moderate hearing loss using obligatory, passively-elicited, cortical auditory evoked potentials (CAEP). High-density EEG elicited by speech stimuli was recorded in adults with hearing loss and age-matched normal hearing controls. Latency, amplitude and source localization of the P1, N1, P2 components of the CAEP were analyzed. Adults with mild-moderate hearing loss showed increases in latency and amplitude of the P2 CAEP relative to control subjects. Current density reconstructions revealed decreased activation in temporal cortex and increased activation in frontal cortical areas for hearing-impaired listeners relative to normal hearing listeners. Participants' behavioral performance on a clinical test of speech perception in noise was significantly correlated with the increases in P2 latency. Our results indicate that changes in cortical resource allocation are apparent in early stages of adult hearing loss, and that these passively-elicited cortical changes are related to behavioral speech perception outcome.

## Introduction

Adults with hearing impairment have been shown to exhibit concomitant deficiencies in cognitive performance (see Craik, 2007; Tun et al., 2012, for a review). A possible reason for this interaction between hearing loss (HL) and cognition may be due to an increase in cognitive load as greater attention is devoted to auditory signals in hearing impairment. For instance, when hearing-impaired adults allocate cognitive processing strategies to understand a degraded incoming auditory signal, the increased load at a basic processing level may detract from later cognitive performance downstream (Pichora-Fuller et al., 1995; Pichora-Fuller and Singh, 2006). As a result, cognitive processes such as memory and executive function are adversely affected in hearing impairment (Arlinger et al., 2009; Lunner et al., 2009; Rönnberg et al., 2010, 2011a,b; Lin, 2011; Rudner et al., 2012; Lin, 2013).

Studies using functional neuroimaging, neural models, and behavioral measures have demonstrated a strong relationship between auditory cortical integrity and the processing of challenging auditory information, such as degraded signals and complex speech in individuals with HL (Wingfield et al., 2006; Harris et al., 2009; Miller and Wingfield, 2010; Peelle et al., 2010a,b, 2011; Wong et al., 2010).

Recent research has shown a compelling correlation between degree of HL severity and all-cause dementia (including Alzheimer's disease), suggesting that increases in auditory deprivation may subsequently influence overall cognitive decline (Lin, 2011, 2012, 2013; Lin et al., 2011a,b). Lin et al. (2011a; Lin, 2013) discuss the decrease in cognitive reserve accompanying HL as a possible mechanism for the link between HL and dementia. Cognitive or neural reserve reflects the ability of the brain to compensate for the deleterious effects of sensory deprivation through the recruitment of alternative or additional brain networks to perform a specific task (Boyle et al., 2008). Sensory deprivation, as in HL, appears to tax the brain by altering normal resource allocation, thereby affecting neural reserve and cognitive performance. Given the relationship between degree of HL and cognitive decline, there appears to be a clear need for systematically examining changes in cortical resource allocation as HL progresses in severity from mild to profound, and to determine whether these changes are apparent at early stages of cortical auditory processing. Electroencephalography (EEG) is a useful measure to examine cortical changes associated with HL due to its non-invasive nature, widespread use in clinical settings and high temporal resolution important in measures of auditory processing.

In this study, we examined cortical re-organization resulting from HL in adult listeners with mild-moderate sensorineural hearing impairment using high-density EEG. We evaluated obligatory, passively-elicited P1, N1, and P2 components of the cortical auditory evoked potential (CAEP) using source localization. We correlated CAEP changes with performance on a clinical test of speech perception in noise to better understand the impact of cortical changes in early stages of hearing decline.

## Methods

### Participants

**Figure 1:**
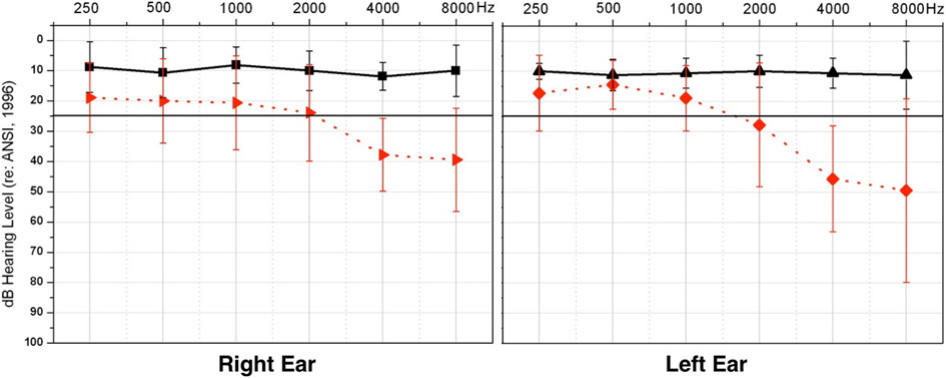
**Average pure tone thresholds across clinical test frequencies (X-axis) for right and left ears, respectively**. Intensity of frequency presentation level is shown on the Y-axis. The normal hearing group (NH) thresholds are depicted in solid black, and the hearing loss (HL) group thresholds in dashed red. Vertical black bars indicate standard deviation. The solid black line illustrates the criterion for normal hearing, at 25 dB HL.

Adults between the ages of 37 to 68 years participated in this study (*n* = 17). Subjects were recruited using fliers and recruitment letters. Consent was obtained through documentation approved by the University of Colorado at Boulder Institutional Review Board. Hearing acuity was measured using standard clinical audiometric procedures. Normal hearing (NH) thresholds [below 25 dB Hearing Level (HL)] for frequencies ranging from 0.25–8 kHz were observed for eight of the participants (*M* = 50.5 years, *SD* = ± 6.2 years), while the remaining nine demonstrated HL (*M* = 56.9 years, *SD* = ± 8.9 years). The HL group showed, on average, NH from 0.25 through 1 kHz and a mild-to-moderate sensorineural HL bilaterally from 2 to 8 kHz. Mean threshold audiograms for the two groups are shown in Figure 1. Participants in the HL group had received no clinical intervention, and many were unaware of their HL at the time of enrollment, consistent with the mild nature of their HL, and suggesting that their HL might have been fairly recent. Participants reported no history of neurological impairment. The NH group and HL group showed no significant difference in age between groups [*t*_(15)_ = −1.69, *p* = 0.537].

### Speech perception in noise

The QuickSIN™, a clinical measure of auditory threshold for sentences in background noise, was used to determine acuity of speech perception in background noise (Killion et al., 2004). Stimuli were presented via a speaker placed at 0° azimuth. Standard clinical testing procedures were used: Listeners were instructed to repeat two sentence lists, consisting of six sentences each, presented at 65 dB HL. Background noise was increased for each consecutive sentence in 5 dB increments, so that the signal-to-noise ratio (SNR) began at 25 dB and ended at 0 dB for the last sentence. The SNR score from the two lists was averaged for each listener, providing the level necessary for each individual to correctly repeat 50% of the key words in each sentence. The lower the SNR score, the greater the level of background noise that could be tolerated by the listener, and the better the performance.

### EEG auditory stimuli

Participants were presented with a nonsense speech syllable, /ba/, at a level of at 65 dB HL, via two speakers placed at 45° angles in relation to the subject (Sharma et al., 2005). Stimuli were presented at a similar intensity level to all subjects consistent with previous studies examining cortical functioning in HL listeners (e.g., Harkrider et al., 2009; Bertoli et al., 2011; Peelle et al., 2011). Subjects were asked to ignore the stimulus while watching a movie, with the sound off and subtitles on, to ensure that participants remained awake (Sharma et al., 2005). Each /ba/ stimulus was 90 ms in duration and was presented at an inter-stimulus interval of 610 ms. One block of 1200 sweeps was collected per subject.

### EEG recording and analyses

Participants were fit with a 128-channel electrode net (Electrical Geodesic, Inc.) and seated in a reclining chair in an electro-magnetically shielded sound booth. Auditory stimuli were presented via stimulus software E-Prime 2.0. The recording sampling rate was 1 kHz, with a band-pass filter of 0.1–200 Hz.

EEG topographic map analysis was completed offline using Net Station 4 (Electrical Geodesic, Inc.). A two-dimensional voltage map was generated for each group grand average waveform for each of the three obligatory CAEP peak components (P1, N1, P2). Regions of interest (ROI) were identified based on the greatest group differences for each CAEP component. Four ROIs were determined to be present: the frontal region, central region, the left frontal hemisphere (LH), and the right frontal hemisphere (RH). Individual EEG data was then exported from Net Station and imported into the EEGLAB toolbox (Delorme and Makeig, 2004) supported by MatLab (The MathWorks®, Inc., 2010). Epoched data was baseline corrected to the pre-stimulus interval of 100 ms and initial artifact rejection performed at ±100 μ V. The sampling rate was down-sampled from 1 kHz to 250 Hz in order to decrease processing time, resulting in a change of the post-stimulus time to 592 ms. Concatenated EEG sweeps were then pruned using an independent component analysis (ICA) statistical procedure (Debener et al., 2006, 2008). Additional artifact such as ocular and other extraneous muscle movement identified as separate components were removed from the data. CAEP waveform peak components were visually identified and averaged after this step. For each subject, three electrodes were then grand averaged in each ROI, except for the central ROI where we averaged across four electrodes. Latency and amplitude values were determined for each participant CAEP waveform. All peak component amplitudes (P1, N1, P2) were measured from baseline to peak, or the midpoint of broad peaks. Latencies were chosen at the highest amplitude of the peak, or the midpoint of broad, flat peaks. Planned statistical comparisons were performed on the CAEP latency and amplitude components averaged within each ROI to determine significant differences between groups.

### Current density reconstructions

ICA on concatenated EEG sweeps was performed to remove artifact, increase signal to noise ratio, and identify underlying components to be sourced. ICA results in multiple temporally independent components that underlie the evoked potential and are fixed in the spatial domain (Makeig et al., 1997; Delorme et al., 2012). These components allow for precise generator localization when used in cortical source modeling (Makeig et al., 2004; Hine and Debener, 2007; Debener et al., 2008). Concatenated EEG sweeps were pruned, as previously described, using ICA in order to remove noise artifact (Debener et al., 2006, 2008). This first pruning was followed by a second pruning to identify major components making up each CAEP peak component. Only independent components that accounted for the greatest percent variance underlying a CAEP peak of interest (P1, N1, P2) were retained for source localization analysis, or current density reconstruction (CDR). The individually pruned waveforms were grand-averaged for the NH and HL groups and exported into CURRY® Scan 7 Neuroimaging Suite (Compumedics Neuroscan™) for CDR. In CURRY®, another ICA was performed on each group average, and only components showing a SNR of at least 2.0 accepted.

CDR was performed separately for each CAEP peak component using sLORETA. Standardized low-resolution brain electromagnetic tomography (sLORETA) is a statistical procedure that estimates a focal CDR with zero localization error using actual source and measurement variance (Pascual-Marqui, 2002; Grech et al., 2008). The selected head model utilized for source modeling consisted of the standardized boundary element method (BEM) (Fuchs et al., 2002). A color scale corresponding to the intensity of cortical activation, as estimated by sLORETA, illustrates the CDR on an average magnetic resonance image (MRI) consisting of 100 people.

## Results

### Auditory evoked potentials

Based on the two-dimensional voltage maps for both groups and group differences between the waveforms, four ROIs were determined in the frontal, central, left frontal hemispheric (LH), and right frontal hemispheric regions (RH). Three obligatory CAEP components elicited by the speech sound were evaluated: the P1 (occurring at approximately 70 ms), N1 (at approximately 100 ms), and P2 (at approximately 180 ms). Group differences for the amplitude and latency of each component were analyzed using a One-Way ANOVA, and planned *post-hoc* comparisons were made between the groups at each ROI.

**Figure 2:**
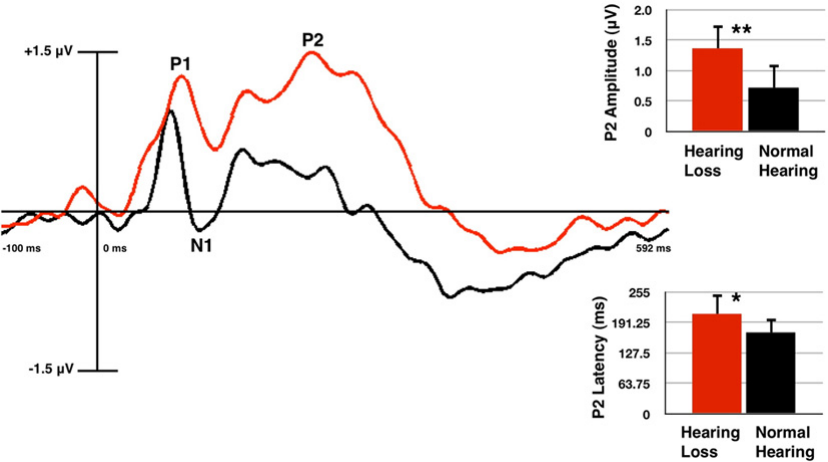
**Grand averaged cortical auditory evoked potentials (CAEPs) from the frontal region of interest (ROI) for the normal hearing (NH, in black) and hearing loss (HL, in red) groups**. P2 amplitude is significantly higher and P2 latency is significantly longer in the HL group as shown in the waveform and mean amplitude bar graphs. Two asterisks indicate significance at *p* < 0.01, one asterisk indicates significance at *p* < 0.05. Vertical bars on the graph show standard deviation.

P2 amplitude was found to be significantly larger in the HL group (relative to the NH group) for the frontal ROI [*F*_(1, 60)_ = 8.7, *p* = 0.005], the central ROI [*F*_(1, 60)_ = 14.97, *p* = 0.000], and the LH ROI [*F*_(1, 60)_ = 8.856, *p* = 0.004], but not at the RH ROI [*F*_(1, 60)_ = 3.621, *p* = 0.062]. P2 latency was found to be significantly longer for the HL group in the frontal ROI [*F*_(1, 60)_ = 5.34, *p* = 0.024], but not the central [*F*_(1, 60)_ = 0.783, *p* = 0.380], LH [*F*_(1, 60)_ = 1.054, *p* = 0.309], or RH [*F*_(1, 60)_ = 3.832, *p* = 0.055] ROIs. P1 amplitude did not differ significantly between groups in any ROI [frontal: *F*_(1, 60)_ = 2.149, *p* = 0.148; central: *F*_(1, 60)_ = 3.715, *p* = 0.059; LH: *F*_(1, 60)_ = 2.446, *p* = 0.123; RH: *F*_(1, 60)_ = 1.661, *p* = 0.202]. P1 latency showed no significant difference [frontal: *F*_(1, 60)_ = 1.163, *p* = 0.285; central: *F*_(1, 60)_ = 0.234, *p* = 0.630; LH: *F*_(1, 60)_ = 0.295, *p* = 0.589; RH: *F*_(1, 60)_ = 0.251, *p* = 0.618]. Similarly, the N1 component did not differ significantly between groups in amplitude [frontal: *F*_(1, 60)_ = 3.685, *p* = 0.060; central: *F*_(1, 60)_ = 0.362, *p* = 0.549; LH: *F*_(1, 60)_ = 3.322, *p* = 0.073; RH: *F*_(1, 60)_ = 0.042, *p* = 0.838], or latency [frontal: *F*_(1, 60)_ = 2.409, *p* = 0.126; central: *F*_(1, 60)_ = 0.020, *p* = 0.887; LH: *F*_(1, 60)_ = 1.625, *p* = 0.207; RH: *F*_(1, 60)_ = 0.851, *p* = 0.360]. Figure 2 shows the grand average waveforms from the frontal ROI, with mean amplitude bar graphs depicting the significantly larger P2 amplitude and longer P2 latency for the HL group compared to the NH group.

It should be noted that we presented the auditory stimuli at a comfortably loud conversational level for our participants. The /ba/ stimulus is comprised of spectral energy occurring mainly in the low-mid frequency region (0.5–2 kHz) (Sharma et al., 2002), and the HL listeners demonstrated average thresholds that were within the normal range at these frequencies. There was an average difference of approximately 10 dB HL between thresholds for the HL and NH group in the 0.5–2 kHz range, therefore, some HL listeners may have heard the stimuli at a sensation level (SL) that was, on average, 10 dB lower than for NH subjects. However, it is important to note that it is a well-established finding that CAEP amplitude decreases with lower intensity level for both NH and HL listeners (Bertoli et al., 2011), while the results of this study show increased P2 amplitude for the HL listeners. That is, if results were influenced by the decreased SL for HL listeners, we would have expected to observe a corresponding decrease in P2 amplitude for HL compared with NH listeners rather than a larger P2 amplitude for HL listeners (Figure 2). Furthermore, our results are consistent with those of Bertoli et al. (2011) and Harkrider et al. (2009), who reported larger P2 amplitudes for adults with mild-moderate HL compared with those for control subjects.

### Current density reconstructions

**Figure 3:**
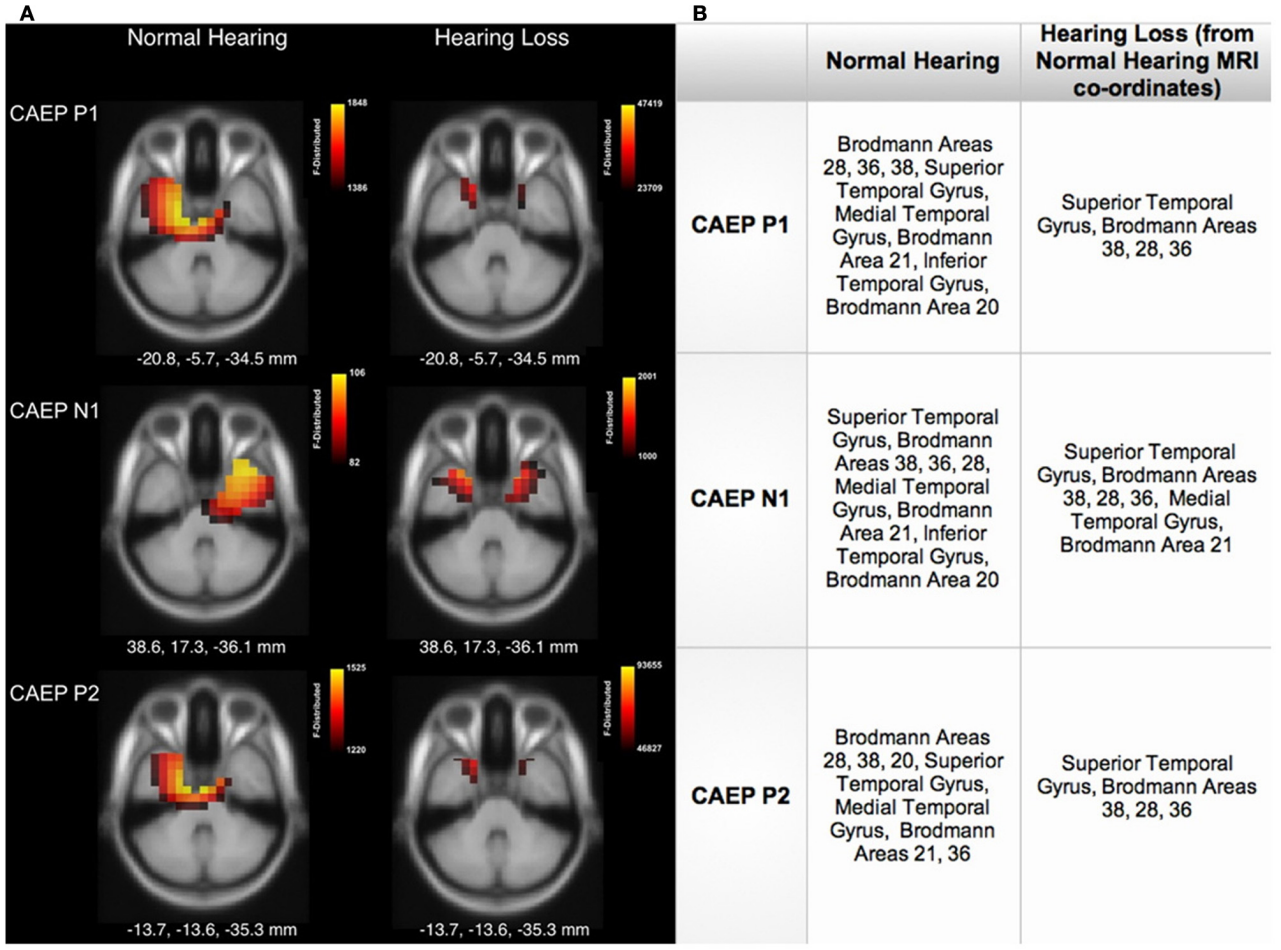
**(A)** Current density reconstructions (CDR) showing cortical activation at the P1, N1, and P2 CAEP peak components on axial MRI slices for the normal hearing (NH) and hearing loss (HL) groups. The scale of the F Distribution is shown in the upper right corner ranging from red to yellow (yellow is highest level of activation), and the Montreal Neurological Institute (MNI) coordinates are listed below each MRI slice. **(B)** A table describing activated anatomical cortical areas for the CAEP components for each group, listed in approximate order of highest level of activation.

Cortical source localization, or CDR, was conducted using the sLORETA algorithm provided by CURRY Scan 7 Neuroimaging Suite for the three CAEP peak components (Figure 3A). The activations were superimposed on an average MRI (axial slice view) and the MNI co-ordinates are shown beneath each slice. The scale of the F distribution, indicating the strength of the activations, is also shown. Figure 3A shows axial views of the CDR. For NH listeners, as seen in Figure 3A, the P1, N1, and P2 CAEP components activated temporal cortical regions including superior temporal gyrus (STG) and inferior temporal gyrus (ITG). Responses for the P1 and P2 components were relegated to the left hemisphere (LH), likely due to our use of a speech syllable (Stefanatos et al., 2008). See Figure 3B for a table describing the main activated regions. Cortical activation by speech stimuli in regions of temporal cortex is consistent with fMRI neuroimaging and intracranial electrocorticographic studies using speech stimuli (Stefanatos et al., 2008; Pasley et al., 2012). In contrast, for the HL group, clearly decreased activation of auditory areas such as STG and MTG within temporal cortex was apparent (see Figure 3A).

**Figure 4:**
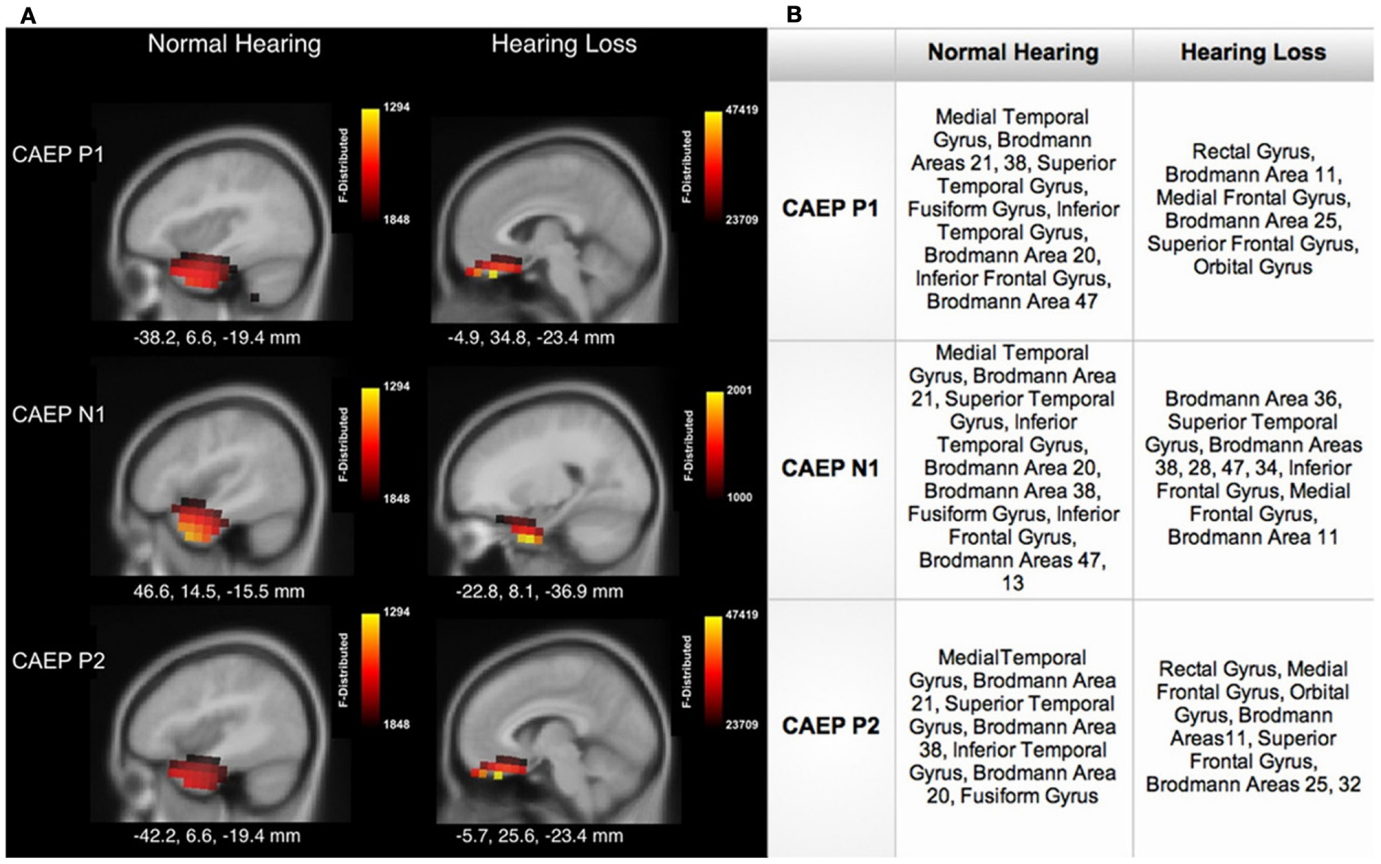
**(A)** Current density reconstructions (CDR) showing cortical activation at the P1, N1, and P2 CAEP peak components on sagittal MRI slices for the normal hearing (NH) and hearing loss (HL) groups. The scale of the F Distribution is shown in the upper right corner ranging from red to yellow (yellow is highest level of activation), and the Montreal Neurological Institute (MNI) coordinates are listed below each MRI slice. **(B)** A table describing activated anatomical cortical areas for the CAEP components for each group, listed in approximate order of highest level of activation.

Figure 4 shows sagittal views for the CDR. Consistent with the axial views shown in Figure 3A, as seen in Figure 4A, NH listeners showed activation of temporal cortical areas including STG and ITG. Conversely, for HL listeners, cortical responses to speech stimuli were localized to frontal cortex, in medial frontal gyrus (MFG), inferior frontal gyrus (IFG), and Brodmann Area 11 (BA 11). See Figure 4B for a table describing the main areas of activation. Frontal cortical activation was clearly the largest for the P1 and P2 CAEP components (Figure 4A).

### Speech perception in noise

**Figure 5:**
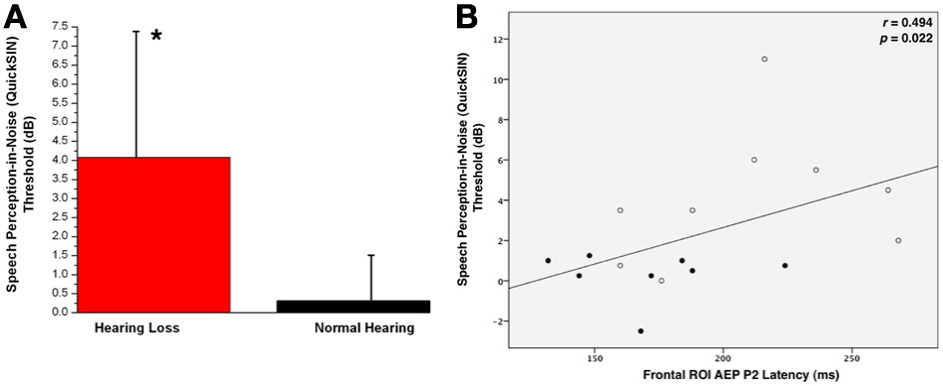
**(A)** Mean QuickSIN™ scores for normal hearing (NH, in black) and hearing loss (HL, in red) groups. Standard deviations are shown as vertical bars. One asterisk reflects a significant difference at *p* < 0.05. **(B)** The correlation of the CAEP P2 component latency as a function of QuickSIN™ scores. The Spearman's rank order correlation coefficient value and significance level are indicated in the right upper corner.

Behavioral testing of speech perception in noise acuity was measured for both groups using the QuickSIN™ clinical test (Killion et al., 2004). The higher the SNR score, the louder the signal has to be in order for the listener to perceive speech. As shown in Figure 5A, the HL group required the signal to be, on average, almost four decibels higher than the background noise for correct perception. Due to the non-parametric distribution of individual QuickSIN™ scores, a Mann-Whitney U Test was calculated to determine statistical significance between the groups (*U* = 10.5, *Z* = −2.46, *p* = 0.014). This difference in performance has been found in similar studies with NH listeners and listeners with HL (Killion et al., 2004; Wilson et al., 2007).

QuickSIN scores were correlated with P2 latency and amplitude. All participants were included in the correlation, as HL can be considered a gradual decrease in threshold starting at 0 dB HL. Frontal ROI P2 latency showed a significant positive correlation with speech performance in background noise (*r* = 0.494, *p* = 0.022), suggesting that increases in P2 latency were associated with greater difficulty in perceiving speech in noise. We did not see a significant correlation between QuickSIN™ scores and P2 amplitude.

### Degree of hearing loss and CAEP P2 amplitude

Frontal P2 amplitude showed a significant positive correlation with high frequency Pure Tone Average (PTA), i.e., the degree of hearing impairment at 2–8 kHz for both ears (right ear: *r* = 0.538, *p* = 0.013, left ear: *r* = 0.474, *p* = 0.027). Thus, as HL increased across participants, there was a corresponding increase in P2 amplitude. No significant correlation was observed between P2 latency and high-frequency PTA.

## Discussion

We examined cortical changes secondary to mild-moderate HL in post-lingually hearing-impaired adults. When tested using speech-evoked EEG in a passive stimulation paradigm, adults with mild to moderate sensorineural HL showed the following distinct cortical changes relative to age-matched NH controls: (1) increased P2 CAEP amplitude and latency, (2) reduced activation in temporal auditory cortical regions, (3) activation of frontal cortical regions in response to auditory stimulation, (4) significantly poorer speech perception in background noise that correlated with increased P2 latency and (5) a significant correlation between increased P2 amplitude and hearing thresholds at high frequencies (2, 4, and 8 kHz). Thus, even in relatively early stages of HL and early stages of auditory processing, adult subjects appear to show significant alterations in cortical activation.

Our finding of increased P2 amplitude for HL listeners is consistent with previous studies, which documented increased P2 CAEP amplitude in older adults who were long-time hearing aid users (Bertoli et al., 2011), and in young adults with mild-moderate HL (Harkrider et al., 2009). Bertoli et al. (2011) reported larger P2 amplitudes for adults with mild-moderate HL who were long-term hearing aid users, and attributed the larger auditory cortical responses in HL adults to an increase in “effortful listening.” It is of further interest to note that larger P2 CAEP amplitudes have been reported after auditory training, possibly indicating increased utilization of auditory memory and perceptual resources (Naatanen and Picton, 1987; Shahin et al., 2003; Ross and Tremblay, 2009; Tong et al., 2009). Along these lines, our finding of an increase in P2 latency is also consistent with previous studies in adults with HL, which suggest that the increased latency reflects inefficient cortical processing as the central auditory system is required to process a degraded and/or challenging signal (Harkrider et al., 2005, 2009; Ross et al., 2007).

Aging has also been reported as a factor in increased P2 amplitude and latency, possibly due to decreased central inhibition. However, we included age-matched, NH listeners, making it unlikely that aging solely accounts for the differences in P2 amplitude and latency seen for the HL group (Harkrider et al., 2006; Ceponiene et al., 2008). Furthermore, Harkrider et al. (2009) observed increased P2 amplitude and latency in young adults with mild-moderate HL in response to nonsense speech syllables, suggesting that higher-order auditory processing is affected by auditory deprivation and not age alone, though an interaction between age and HL is likely. In the case of older listeners with HL, reduced central inhibition via an interaction between aging and HL may result in increased P2 amplitude (Dustman et al., 1996; Syka, 2002). Our results also showed a significant increase in P2 amplitude for the HL group relative to the NH groups over the LH but not the right hemisphere (RH). Given our use of a speech stimulus, the larger P2 amplitude in the HL group over the LH may be due to more active role of the LH in processing of speech information combined with a lack of inhibition due to HL (Syka, 2002; Stefanatos et al., 2008).

A major finding in our study was that listeners with mild-moderate sensorineural HL showed significant cortical re-organization. Current density reconstructions via sLORETA revealed that HL listeners showed decreased activation of auditory cortical areas (STG and MTG) relative to NH listeners (Figure 3A) and showed activation of frontal cortical regions (e.g., IFG, MFG, SFG) in response to passive auditory stimulation (Figure 4A). This change in cortical activation from temporal regions to frontal regions indicates a possible re-allocation of cortical processing in response to auditory stimuli, likely as a compensatory effect of HL. The finding of a shift of the auditory response to frontal areas is consistent with the fMRI studies of Peelle et al. (2010a, 2011) and Wingfield and Grossman (2006), who showed lower amounts of gray matter volume in temporal cortices in adults with HL, as well as greater activation in frontal cortices in response to challenging listening conditions for older adults. This frontal and pre-frontal activation was associated with increased listening effort, as these regions have been traditionally associated with tasks involving working memory and executive function (Collette et al., 2006; Eckert et al., 2008; Liakakis et al., 2011). Overall, our results are consistent with neuroimaging research, which has demonstrated a reliance on frontal regions involved in the cognition and the processing of complex auditory stimuli in older adults (Sharp et al., 2006; Eckert et al., 2008; Tyler et al., 2010; Obleser et al., 2011). Thus, the present results of cortical re-organization in HL adults support recent hypotheses suggestive of an increased cognitive load in hearing impaired listeners, and may provide evidence for the taxation of the reserve of cognitive processes (Pichora-Fuller and Singh, 2006; Lin, 2011, 2012; Lin et al., 2011a,b).

It is surprising, however, that we observed that frontal cortical regions, typically associated with cognitive processing, were engaged in response to a passive auditory task that did not require the participants' attention. This finding suggests that compensatory processing may begin at early stages of central auditory processing in adult-onset HL (Harris et al., 2009; Anderson and Kraus, 2010). Indeed, another form of compensatory plasticity (i.e., recruitment of auditory cortical regions for visual processing) has been observed in adults with mild-moderate HL in whom passively viewed visual stimuli activated temporal cortical regions (Campbell and Sharma, in review). Recent studies have shown similar temporal cortical activation by visual stimuli in deaf adults fitted with cochlear implants (Doucet et al., 2006; Buckley and Tobey, 2011; Sandmann et al., 2012). Visual information becomes of greater importance in HL, especially in watching a speaker's face and lip movements for contextual cues (McCullough et al., 2005; Letourneau and Mitchell, 2011). These findings, taken together with the present results, suggest that increased frontal activation and reduced temporal activation to speech may occur in parallel with increased temporal activation to visual stimuli (likely due to reliance on faces and lipreading in everyday communication), even as early as in mild-moderate HL. Thus, cortical re-allocation during processing of auditory stimuli may result in increased cognitive load that usually occurs in higher-order processing, but that is now occurring for lower-level passive processing, resulting in degraded behavioral outcomes for challenging listening environments (Pichora-Fuller and Singh, 2006; Larsby et al., 2008). It is possible that various training paradigms using speech and music (possibly in conjunction with hearing aid rehabilitation) may allow for re-training of auditory cortices in HL listeners to re-activate normal neural networks during auditory processing (Petersen et al., 2009; Shahin, 2011; Turner et al., 2013).

Hearing loss is most consistently associated with poor outcomes in recognizing speech in background noise, a skill essential for everyday listening (Souza et al., 2007). Consistent with previous research in hearing-impaired adults, our results show that listeners with even mild-to-moderate HL demonstrate a significant deficit when listening to speech in background noise (Dubno, 1984; Vermiglio et al., 2012). HL listeners required a much larger SNR to accurately perceive sentences in noise (Figure 5A). Audibility does not appear to fully account for this decrease in performance (Hällgren et al., 2005; Souza et al., 2007; Léger et al., 2012; Vermiglio et al., 2012). In this study, speech perception in background noise was significantly correlated with increased P2 latency (Figure 5B). This increase in latency is consistent with previous studies suggesting that the increase in auditory processing time (as reflected by the P2 latency increase in the HL group) may be reflective of additional activated cognitive cortical regions, or compensatory cortical circuitry (Ross et al., 2007; Harkrider et al., 2009). In addition, larger P2 CAEP amplitudes were correlated with worse auditory pure tone thresholds at high frequencies (2, 4, and 8 kHz). Given that P2 amplitude has been associated with re-allocation of cognitive resources, (Tremblay et al., 2003; Harkrider et al., 2005, 2009; Tong et al., 2009), it would appear that the degree of cortical re-organization increases with the severity of the HL.

Taken together, the observed increase in P2 CAEP amplitude and latency, decreased activation in temporal areas with increased activation of frontal cortical regions during passive listening, and poorer behavioral outcomes in the HL group, provide evidence of compensatory cortical plasticity occurring in mild-moderate HL (i.e., in early stages of hearing decline). The nature of this plasticity is observed as a re-allocation of cortical resources from temporal auditory areas to frontal cognitive areas, which appear to be recruited to assist with processing of auditory stimuli even at the level of passive listening. Overall, our results are consistent with the hypothesis that HL appears to initiate a process of resource re-allocation, which results in increased cognitive load (Pichora-Fuller et al., 1995; Pichora-Fuller and Singh, 2006; Peelle et al., 2010a,b, 2011; Lin, 2011, 2012, 2013; Lin et al., 2011a,b). Finally, measures of cognitive resource re-allocation in HL, both objective and behavioral, may become increasingly relevant in the clinical setting in order to determine patients at risk for cognitive decline. It would be of interest to determine whether hearing aids, auditory training, or a combination might possibly alleviate this cognitive resource re-allocation as reflected by a possible decrease in frontal activation and return to normal levels of temporal cortical activation (Lunner et al., 2009; Parbery-Clark et al., 2011; Rudner et al., 2012).

## Summary

Our results demonstrate auditory cortical re-organization in the form of decreased temporal activation and increased frontal activation in early stage HL of mild-moderate severity using passively elicited EEG responses. Furthermore, increased latency and amplitude of the P2 component were associated with decreases in speech perception performance and increase in hearing threshold, respectively. Due to the strong relationship between HL and cognitive deficits, such as dementia, that arise later in life, it is important that clinical evaluation of cognitive reserve in HL be included as part of intervention services. Future research should focus on better understanding the relationship between the severity of cognitive re-allocation in relation to severity of HL as well as reversibility of re-organization as a result of intervention with amplification.

### Conflict of interest statement

The authors declare that the research was conducted in the absence of any commercial or financial relationships that could be construed as a potential conflict of interest.

## References

[B1] Anderson S. Kraus N. (2010). Sensory-cognitive interaction in the neural encoding of speech in noise: a review. J. Am. Acad. Audiol. 21, 575–585 10.3766/jaaa.21.9.3 21241645 PMC3075209

[B2] Arlinger S. Lunner T. Lyxell B. Pichora-Fuller M. K. (2009). The emergence of cognitive hearing science. Scand. J. Psychol. 50, 371–384 10.1111/j.1467-9450.2009.00753.x 19778385

[B3] Bertoli S. Probst R. Bodmer D. (2011). Late auditory evoked potentials in elderly long-term hearing-aid users with unilateral or bilateral fittings. Hear. Res. 280, 58–69 10.1016/j.heares.2011.04.013 21569828

[B4] Boyle P. A. Wilson R. S. Schneider J. A. Bienias J. L. (2008). Processing resources reduce the effect of Alzheimer pathology on other cognitive systems. Neurology 70, 1534–1542 10.1212/01.wnl.0000304345.14212.38 18354077 PMC10382255

[B5] Buckley K. A. Tobey E. A. (2011). Cross-modal plasticity and speech perception in pre- and postlingually deaf cochlear implant users. Ear Hear. 32, 2–15 10.1097/AUD.0b013e3181e8534c 20829699

[B6] Ceponiene R. Westerfield M. Torki M. Townsend J. (2008). Modality-specificity of sensory aging in vision and audition: evidence from event-related potentials. Brain Res. 1215, 53–68 10.1016/j.brainres.2008.02.010 18482717

[B7] Collette F. Hogge M. Salmon E. Van der Linden M. (2006). Exploration of the neural substrates of executive functioning by functional neuroimaging. Neuroscience 139, 209–221 10.1016/j.neuroscience.2005.05.035 16324796

[B8] Craik F. I. M. (2007). The role of cognition in age-related hearing loss. J. Am. Acad. Audiol. 18, 539–547 10.3766/jaaa.18.7.2 18236642

[B9] Debener S. Hine J. Bleeck S. Eyles J. (2008). Source localization of auditory evoked potentials after cochlear implantation. Psychophysiology 45, 20–24 10.1111/j.1469-8986.2007.00610.x 17910729

[B10] Debener S. Ullsperger M. Siegel M. Engel A. K. (2006). Single-trial EEG–fMRI reveals the dynamics of cognitive function. Trends Cogn. Sci. 10, 558–563 10.1016/j.tics.2006.09.010 17074530

[B11] Delorme A. Makeig S. (2004). EEGLAB: an open source toolbox for analysis of single-trial EEG dynamics including independent component analysis. J. Neurosci. Methods 134, 9–21 10.1016/j.jneumeth.2003.10.009 15102499

[B12] Delorme A. Palmer J. Onton J. Oostenveld R. Makeig S. (2012). Independent EEG sources are dipolar. PLoS ONE 7:e30135 10.1371/journal.pone.0030135 22355308 PMC3280242

[B13] Doucet M. E. Bergeron F. Lassonde M. Ferron P. Lepore F. (2006). Cross-modal reorganization and speech perception in cochlear implant users. Brain 129(pt 12), 3376–3383 10.1093/brain/awl264 17003067

[B14] Dubno J. R. (1984). Effects of age and mild hearing loss on speech recognition in noise. J. Acoust. Soc. Am. 76, 87–96 10.1121/1.391011 6747116

[B15] Dustman R. E. Emmerson R. Y. Shearer D. E. (1996). Life span changes in electrophysiological measures of inhibition. Brain Cogn. 30, 109–126 10.1006/brcg.1996.0007 8811986

[B16] Eckert M. A. Walczak A. Ahlstrom J. Denslow S. Horwitz A. Dubno J. R. (2008). Age-related effects on word recognition: reliance on cognitive control systems with structural declines in speech-responsive cortex. J. Assoc. Res. Otolaryngol. 9, 252–259 10.1007/s10162-008-0113-3 18274825 PMC2504602

[B17] Fuchs M. Kastner J. Wagner M. Hawes S. Ebersole J. S. (2002). A standardized boundary element method volume conductor model. Clin. Neurophysiol. 113, 702–712 10.1016/S1388-2457(02)00030-5 11976050

[B18] Grech R. Cassar T. Muscat J. Camilleri K. P. Fabri S. G. Zervakis M. (2008). Review on solving the inverse problem in EEG source analysis. J. Neuroeng. Rehabil. 5:25 10.1186/1743-0003-5-2 18990257 PMC2605581

[B19] Hällgren M. Larsby B. Lyxell B. (2005). Speech understanding in quiet and noise, with and without hearing aids. Int. J. Audiol. 44, 574–583 10.1080/14992020500190011 16315448

[B20] Harkrider A. W. Plyler P. N. Hedrick M. S. (2005). Effects of age and spectral shaping on perception and neural representation of stop consonant stimuli. Clin. Neurophysiol. 116, 2153–2164 10.1016/j.clinph.2005.05.016 16043402

[B21] Harkrider A. W. Plyler P. N. Hedrick M. S. (2006). Effects of hearing loss and spectral shaping on identification and neural response patterns of stop-consonant stimuli. J. Acoust. Soc. Am. 120, 915–925 10.1121/1.2204588 16938979

[B22] Harkrider A. W. Plyler P. N. Hedrick M. S. (2009). Effects of hearing loss and spectral shaping on identification and neural response patterns of stop-consonant stimuli in young adults. Ear Hear. 30, 31–42 10.1097/AUD.0b013e31818f359f 19125025

[B23] Harris K. C. Dubno J. R. Keren N. I. Ahlstrom J. B. Eckert M. A. (2009). Speech recognition in younger and older adults: a dependency on low-level auditory cortex. J. Neurosci. 29, 6078–6087 10.1523/JNEUROSCI.0412-09.2009 19439585 PMC2717741

[B24] Hine J. Debener S. (2007). Late auditory evoked potentials asymmetry revisited. Clin. Neurophysiol. 118, 1274–1285 10.1016/j.clinph.2007.03.012 17462945

[B25] Killion M. C. Niquette P. A. Gudmundsen G. I. Revit L. J. Banerjee S. (2004). Development of a quick speech-in-noise test for measuring signal-to-noise ratio loss in normal-hearing and hearing-impaired listeners. J. Acoust. Soc. Am. 116(4 pt 1), 2395–2405 10.1121/1.1784440 15532670

[B26] Larsby B. Hällgren M. Lyxell B. (2008). The interference of different background noises on speech processing in elderly hearing impaired subjects. Int. J. Audiol. 47, S83–S90 10.1080/14992020802301159 19012115

[B27] Léger A. C. Moore B. C. J. Lorenzi C. (2012). Abnormal speech processing in frequency regions where absolute thresholds are normal for listeners with high-frequency hearing loss. Hear. Res. 294, 95–103 10.1016/j.heares.2012.10.002 23104012

[B28] Letourneau S. M. Mitchell T. V. (2011). Gaze patterns during identity and emotion judgments in hearing adults and deaf users of American sign language. Perception 40, 563 10.1068/p6858 21882720 PMC3454476

[B29] Liakakis G. Nickel J. Seitz R. J. (2011). Diversity of the inferior frontal gyrus—a meta-analysis of neuroimaging studies. Behav. Brain Res. 225, 341–347 10.1016/j.bbr.2011.06.022 21729721

[B30] Lin F. R. (2011). Hearing loss and cognition among older adults in the United States. J. Gerontol. A Biol. Sci. Med. Sci. 66, 1131–1136 10.1093/gerona/glr115 21768501 PMC3172566

[B31] Lin F. R. (2012). Hearing loss in older adults: who's listening? JAMA 307, 1147–1148 10.1001/jama.2012.321 22436953 PMC3518399

[B32] Lin F. R. (2013). Hearing loss and cognitive decline in older adults. JAMA Intern. Med. 173, 293 10.1001/jamainternmed.2013.1868 23337978 PMC3869227

[B33] Lin F. R. Ferrucci L. Metter E. J. An Y. Zonderman A. B. Resnick S. M. (2011a). Hearing loss and cognition in the Baltimore Longitudinal Study of Aging. Neuropsychology 25, 763–770 10.1037/a0024238 21728425 PMC3193888

[B34] Lin F. R. Metter E. J. O'Brien R. J. Resnick S. M. Zonderman A. B. Ferrucci L. (2011b). Hearing loss and incident dementia. Arch. Neurol. 68, 214–220 10.1001/archneurol.2010.362 21320988 PMC3277836

[B35] Lunner T. Rudner M. Rönnberg J. (2009). Cognition and hearing aids. Scand. J. Psychol. 50, 395–403 10.1111/j.1467-9450.2009.00742.x 19778387

[B36] Makeig S. Delorme A. Westerfield M. Jung T. P. Townsend J. Courchesne E. (2004). Electroencephalographic brain dynamics following manually responded visual targets. PLoS Biol. 2:e176 10.1371/journal.pbio.0020176 15208723 PMC423146

[B37] Makeig S. Jung T. P. Bell A. J. Ghahremani D. Sejnowski T. J. (1997). Blind separation of auditory event-related brain responses into independent components. Proc. Natl. Acad. Sci. U.S.A. 94, 10979–10984 10.1073/pnas.94.20.10979 9380745 PMC23551

[B38] McCullough S. Emmorey K. Sereno M. (2005). Neural organization for recognition of grammatical and emotional facial expressions in deaf ASL signers and hearing nonsigners. Brain Res. Cogn. Brain Res. 22, 193–203 10.1016/j.cogbrainres.2004.08.012 15653293

[B39] Miller P. Wingfield A. (2010). Distinct effects of perceptual quality on auditory word recognition, memory formation and recall in a neural model of sequential memory. Front. Syst. Neurosci. 4:14 10.3389/fnsys.2010.00014 20631822 PMC2901090

[B40] Naatanen R. Picton T. (1987). The N1 wave of the human electric and magnetic response to sound: a review and an analysis of the component structure. Psychophysiology 24, 375–425 10.1111/j.1469-8986.1987.tb00311.x 3615753

[B41] Obleser J. Meyer L. Friederici A. D. (2011). Dynamic assignment of neural resources in auditory comprehension of complex sentences. Neuroimage 56, 2310–2320 10.1016/j.neuroimage.2011.03.035 21421059

[B42] Parbery-Clark A. Strait D. L. Anderson S. Hittner E. Kraus N. (2011). Musical experience and the aging auditory system: implications for cognitive abilities and hearing speech in noise. PLoS ONE 6:e18082 10.1371/journal.pone.0018082 21589653 PMC3092743

[B43] Pascual-Marqui R. D. (2002). Standardized low-resolution brain electromagnetic tomography (sLORETA): technical details. Methods Find. Exp. Clin. Pharmacol. 24 (Suppl. D), 5–12 12575463

[B44] Pasley B. N. David S. V. Mesgarani N. Flinker A. Shamma S. A. Crone N. E. (2012). Reconstructing speech from human auditory cortex. PLoS Biol. 10:e1001251 10.1371/journal.pbio.1001251 22303281 PMC3269422

[B45] Peelle J. E. Johnsrude I. S. Davis M. H. (2010a). Hierarchical processing for speech in human auditory cortex and beyond. Front. Hum. Neurosci. 4:51 10.3389/fnhum.2010.00051 20661456 PMC2907234

[B46] Peelle J. E. Troiani V. Wingfield A. Grossman M. (2010b). Neural processing during older adults' comprehension of spoken sentences: age differences in resource allocation and connectivity. Cereb. Cortex 20, 773–782 10.1093/cercor/bhp142 19666829 PMC2837088

[B47] Peelle J. E. Troiani V. Grossman M. Wingfield A. (2011). Hearing loss in older adults affects neural systems supporting speech comprehension. J. Neurosci. 31, 12638–12643 10.1523/JNEUROSCI.2559-11.2011 21880924 PMC3175595

[B48] Petersen B. Mortensen M. V. Gjedde A. Vuust P. (2009). Reestablishing speech understanding through musical ear training after cochlear implantation. Ann. N.Y. Acad. Sci. 1169, 437–440 10.1111/j.1749-6632.2009.04796.x 19673820

[B49] Pichora-Fuller M. K. Schneider B. A. Daneman M. (1995). How young and old adults listen to and remember speech in noise. J. Acoust. Soc. Am. 97, 593–608 10.1121/1.412282 7860836

[B50] Pichora-Fuller M. K. Singh G. (2006). Effects of age on auditory and cognitive processing: implications for hearing aid fitting and audiologic rehabilitation. Trends Amplif. 10, 29–59 10.1177/108471380601000103 16528429 PMC4111543

[B53] Rönnberg J. Rudner M. Lunner T. Zekveld A. (2010). When cognition kicks in: working memory and speech understanding in noise. Noise Health 12, 263 10.4103/1463-1741.70505 20871181

[B51] Rönnberg J. Danielsson H. Rudner M. Arlinger S. Sternang O. Wahlin A. (2011a). Hearing loss is negatively related to episodic and semantic long-term memory but not to short-term memory. J. Speech Lang. Hear. Res. 54, 705–726 10.1044/1092-4388(2010/09-0088) 20884779

[B52] Rönnberg J. Rudner M. Lunner T. (2011b). Cognitive hearing science: the legacy of Stuart Gatehouse. Trends Amplif. 15, 140–148 10.1177/1084713811409762 21606047 PMC4040830

[B54] Ross B. Fujioka T. Tremblay K. L. Picton T. W. (2007). Aging in binaural hearing begins in mid-life: evidence from cortical auditory-evoked responses to changes in interaural phase. J. Neurosci. 27, 11172–11178 10.1523/JNEUROSCI.1813-07.2007 17942712 PMC6673023

[B55] Ross B. Tremblay K. (2009). Stimulus experience modifies auditory neuromagnetic responses in young and older listeners. Hear. Res. 248, 48–59 10.1016/j.heares.2008.11.012 19110047 PMC2668103

[B56] Rudner M. Lunner T. Behrens T. Thorén E. S. Rönnberg J. (2012). Working memory capacity may influence perceived effort during aided speech recognition in noise. J. Am. Acad. Audiol. 23, 577–589 10.3766/jaaa.23.7.7 22967733

[B57] Sandmann P. Dillier N. Eichele T. Meyer M. Kegel A. Pascual-Marqui R. D. (2012). Visual activation of auditory cortex reflects maladaptive plasticity in cochlear implant users. Brain 135, 555–568 10.1093/brain/awr329 22232592

[B58] Shahin A. J. (2011). Neurophysiological influence of musical training on speech perception. Front. Psychol. 2:126 10.3389/fpsyg.2011.00126 21716639 PMC3115576

[B59] Shahin A. Bosnyak D. J. Trainor L. J. Roberts L. E. (2003). Enhancement of neuroplastic P2 and N1c auditory evoked potentials in musicians. J. Neurosci. 23, 5545–5552 12843255 10.1523/JNEUROSCI.23-13-05545.2003 PMC6741225

[B60] Sharma A. Dorman M. F. Spahr A. J. (2002). A sensitive period for the development of the central auditory system in children with cochlear implants: implications for age of implantation. Ear Hear. 23, 532–539 10.1097/01.AUD.0000042223.62381.01 12476090

[B61] Sharma A. Martin K. Roland P. Bauer P. (2005). P1 latency as a biomarker for central auditory development in children with hearing impairment. J. Am. Acad. Audiol. 16, 564–573 10.3766/jaaa.16.8.5 16295243

[B62] Sharp D. J. Scott S. K. Mehta M. A. Wise R. J. S. (2006). The neural correlates of declining performance with age: evidence for age-related changes in cognitive control. Cereb. Cortex 16, 1739–1749 10.1093/cercor/bhj109 16407479

[B63] Souza P. E. Boike K. T. Witherell K. Tremblay K. (2007). Prediction of speech recognition from audibility in older listeners with hearing loss: effects of age, amplification, and background noise. J. Am. Acad. Audiol. 18, 54–65 10.3766/jaaa.18.1.5 17252958

[B64] Stefanatos G. A. Joe W. Q. Aguirre G. K. Detre J. A. (2008). Activation of human auditory cortex during speech perception: effects of monaural, binaural, and dichotic presentation. Neuropsychologia 46, 301–315 10.1016/j.neuropsychologia.2007.07.008 18023460

[B65] Syka J. (2002). Plastic changes in the central auditory system after hearing loss, restoration of function, and during learning. Physiol. Rev. 82, 601–636 10.1152/physrev.00002.2002 12087130

[B66] Tong Y. Melara R. D. Rao A. (2009). P2 enhancement from auditory discrimination training is associated with improved reaction times. Brain Res. 1297, 80–88 10.1016/j.brainres.2009.07.089 19651109

[B67] Tremblay K. L. Piskosz M. Souza P. (2003). Effects of age and age-related hearing loss on the neural representation of speech cues. Clin. Neurophysiol. 114, 1332–1343 10.1016/S1388-2457(03)00114-7 12842732

[B68] Tun P. A. Williams V. A. Small B. J. Hafter E. R. (2012). The effects of aging on auditory processing and cognition. Am. J. Audiol. 21, 344–350 10.1044/1059-0889(2012/12-0030) 23233520

[B69] Turner J. G. Parrish J. L. Zuiderveld L. Darr S. Hughes L. F. Caspary D. M. (2013). Acoustic experience alters the aged auditory system. Ear Hear. 34, 151–159 10.1097/AUD.0b013e318269ca5b 23086424 PMC3740174

[B70] Tyler L. K. Shafto M. A. Randall B. Wright P. Marslen-Wilson W. D. Stamatakis E. A. (2010). Preserving syntactic processing across the adult life span: the modulation of the frontotemporal language system in the context of age-related atrophy. Cereb. Cortex 20, 352–364 10.1093/cercor/bhp105 19505991 PMC2803734

[B71] Vermiglio A. J. Soli S. D. Freed D. J. Fisher L. M. (2012). The relationship between high-frequency pure-tone hearing loss, hearing in noise test (HINT) thresholds, and the articulation index. J. Am. Acad. Audiol. 23, 779–788 10.3766/jaaa.23.10.4 23169195

[B72] Wilson R. H. McArdle R. A. Smith S. L. (2007). An evaluation of the BKB-SIN, HINT, QuickSIN, and WIN materials on listeners with normal hearing and listeners with hearing loss. J. Speech Lang. Hear. Res. 50, 844–856 10.1044/1092-4388(2007/059) 17675590

[B73] Wingfield A. Grossman M. (2006). Language and the aging brain: patterns of neural compensation revealed by functional brain imaging. J. Neurophysiol. 96, 2830–2839 10.1152/jn.00628.2006 17110737

[B74] Wingfield A. McCoy S. L. Peelle J. E. Tun P. A. Cox L. C. (2006). Effects of adult aging and hearing loss on comprehension of rapid speech varying in syntactic complexity. J. Am. Acad. Audiol. 17, 487–497 10.3766/jaaa.17.7.4 16927513

[B75] Wong P. C. Ettlinger M. Sheppard J. P. Gunasekera G. M. Dhar S. (2010). Neuroanatomical characteristics and speech perception in noise in older adults. Ear Hear. 31, 471–479 10.1097/AUD.0b013e3181d709c2 20588117 PMC2919052

